# Prevalence of major infectious diseases in backyard chickens from rural markets in Morocco

**DOI:** 10.14202/vetworld.2023.1897-1906

**Published:** 2023-09-21

**Authors:** Asma Fagrach, Oumaima Arbani, Oumaima Karroute, Fatima Zahra El-Ftouhy, Faouzi Kichou, Mohammed Bouslikhane, Siham Fellahi

**Affiliations:** 1 Department of Pathology and Veterinary Public Health, Institut Agronomique et Vétérinaire Hassan II, BP 6202, Rabat, Morocco; 2 Laboratory of Biochemistry, Environment and Agri-food, Faculty of Science and Technology Mohammedia, University Hassan II, Casablanca, Morocco

**Keywords:** avian diseases, backyard chickens, low pathogenic avian influenza H9N2, Newcastle disease, risk factors, rural markets

## Abstract

**Background and Aim:**

Raising backyard chickens is a common practice in Morocco, mainly in rural or periurban areas. Constraints due to devastating avian diseases have been recognized as a major limiting factor in backyard poultry production. Consequently, these flocks could potentially be implicated as reservoirs for poultry diseases. However, there is a considerable lack of information on disease prevalence in this production system, and the risk represented by these small flocks remains under debate. This study aimed to estimate the seroprevalence and identify related risk factors of a range of bacterial and viral pathogens of outstanding importance for the economy and public health in backyard poultry in Morocco.

**Materials and Methods:**

A total of 712 sera samples and 258 cloacal swabs were collected from 712 backyard chickens from 15 rural markets in the Khemisset and Skhirat-Temara provinces. None of the sampled chickens received any vaccination. Sera samples were screened for antibodies against Newcastle disease virus (NDV) and low pathogenic avian influenza H9N2 subtype (LPAI H9N2) using a hemagglutination-inhibition test, against bursal infectious disease virus (IBDV) and infectious bronchitis virus (IBV) using enzyme-linked immunosorbent assay, and against *Mycoplasma gallisepticum* (MG) and *Mycoplasma synoviae* (MS) using a rapid serum agglutination test. Swab samples were compiled into 86 pools and submitted for molecular detection using real-time reverse-transcription–polymerase chain reaction (RT-PCR).

**Results:**

The seroprevalences in backyard chickens for NDV, LPAI H9N2, IBDV, IBV, MG, and MS were 52.1% (371/712), 63.5% (452/712), 84.7% (603/712), 82.2% (585/712), 58% (413/712), and 74.8% (533/712), respectively. Based on the RT-PCR results, 2.3% (2/86), 62.8% (54/86), 2.3% (2/86), 63.9% (55/86), 40.7% (35/86), and 29.1% (25/86) of the pools were positive for NDV, H9N2 LPAI, IBDV, IBV, MG, and MS, respectively. Multiple coinfections (H9N2-IBV-MG), (H9N2-IBV-MS), or (IBV-MG-MS) were observed in 15.1%, 8.5%, and 8.5% of the tested samples, respectively.

**Conclusion:**

The results show that backyard chicken flocks and rural markets have the potential to serve as reservoirs or amplifiers for poultry pathogens and could pose a risk to the commercial poultry sector. This highlights the need for a comprehensive and adapted vaccination plan for backyard chickens, and extension of efforts to increase flock owners’ awareness of avian diseases and incite the implementation of biosecurity measures at the farm level.

## Introduction

In recent decades, the Moroccan poultry sector has undergone remarkable expansion, with the country currently being a significant contributor to the production of chicken meat and eggs on the African continent. According to the Interprofessional Federation of the Poultry Sector [1], this industry generates approximately $304 million annually, with more than 425 million broilers and 5.5 billion eggs produced each year. Despite the sector’s potential for growth, it continues to face significant instability and numerous challenges. These challenges mainly include fluctuations in the price of raw materials and hydrocarbons. In addition to market uncertainties, the emergence of avian diseases poses a substantial problem for poultry enterprises, as it can lead to significant economic losses resulting from reduced egg production, increased feed conversion, and high mortality rates. As a result, industry professionals expend substantial financial resources each year on preventive measures such as vaccination, diagnosis, and treatments.

Despite the above, backyard poultry farming in Morocco still occupies an important and promising position as, it can contribute to food security, income improvement, women’s empowerment, and alleviation of poverty [2] as well as to the capacity of the rural population to deal with different recurrent crises such as poor harvests, drought, and climatic disasters. Backyard poultry farming allows vulnerable families to ensure a constant source of animal proteins and to generate income through the marketing of birds and eggs [3]. Moreover, there has been a significant increase in backyard poultry farming in recent years due to the growing consumer demand for organic and free-range poultry products [4]. This expansion, coupled with poor biosecurity and hygiene practices, makes these flocks a potential reservoir of avian pathogens that can be devastating and affect poultry in the commercial sector [5]. Furthermore, rural markets, where backyard birds are transported for sale, could play a major role in facilitating the emergence and spread of avian diseases such as avian influenza and Newcastle disease [6, 7]. Some of these diseases can be zoonotic with fatal consequences for poultry and humans, including salmonella infection and campylobacteriosis [8, 9]. Despite the potential threat of the emergence and spread of avian diseases in backyard poultry in Morocco, to date there is little evidence of their occurrence in backyard poultry flocks. Therefore, it is of interest to investigate whether these diseases are spread among backyard poultry flocks, especially in the Rabat-Sale-Kenitra region, which plays a significant role in the Moroccan poultry industry due to the large number and high density of poultry farms installed in this area.

This study aimed to estimate the prevalence of a selected group of avian pathogens among backyard chickens at rural markets in Khemisset and Temara provinces to obtain more information on the spread of these pathogens and explore the potential transmission risk for the industrial sector.

## Materials and Methods

### Ethical approval

This study does not require the approval of the Institutional Ethics Committee as live animals were not used in the study.

### Study period and location

**Figure-1:**
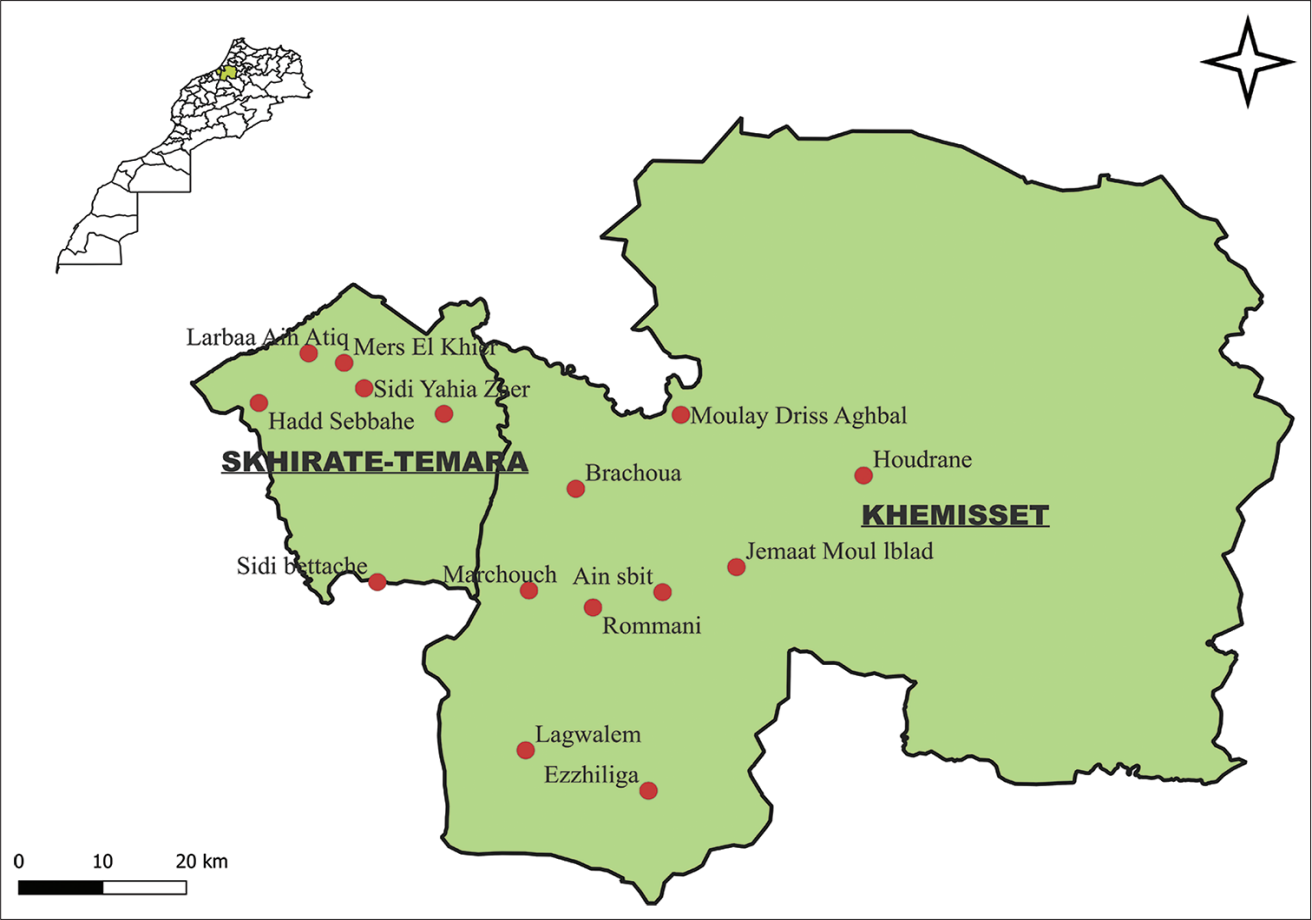
Geographical distribution of the 15 sampled rural markets [Source: The map was generated using QGIS version 2.26.3].

The study was conducted from February 2021 to June 2022. We selected the weekly rural markets of Khemisset and Temara provinces as sampling sites. A weekly rural market is held once a week, during which backyard poultry flock owners and traders market weekly live or slaughtered poultry, among other farm products. Khemisset and Skhirate-Temara provinces have 36 weekly rural markets. Most of the rural markets are generally poorly structured and unhygienic, and poultry of different species are kept together due to space constraints. We targeted 15 major rural markets in the area (10 in Khemisset and 5 in Temara). The global positioning system coordinate data of each rural market were collected using Google Maps™ (https://www.google.com/maps) and entered into a digitalized map of the Rabat-Sale-Kenitra region. We used a geographic information system program (QGIS version 3.2, https://download.qgis.org/qgisdata/QGIS-Website/live/html/fr/site/index.html) to prepare a map showing the spatial distribution of the sampled rural markets (Figure-1).

### Sampling procedure

Using a convenience sampling technique, we collected blood samples and cloacal swabs at slaughter from 712 apparently healthy local chickens >2 months of age. Approximately 5 mL of blood was collected aseptically from each chicken using a clot activator vacutainer and allowed to clot under normal atmospheric conditions. Cloacal swabs were obtained using Dacron swabs. A total of 712 blood samples and 258 swabs were obtained. Samples were labeled and transported in a cool box to the Avian Pathology Unit at Institut Agronomique et Vétérinaire Hassan II in Rabat. Sera were harvested into properly labeled 2-mL Eppendorf tubes and stored with the cloacal swabs at −20°C until analysis. In addition to collecting samples, we also conducted interviews with chicken owners to gather information about the health status of their chickens over the past 6 months. We conducted the interviews using a structured questionnaire administered in face-to-face meetings, with questions focused on symptoms related to the respiratory, digestive, and nervous systems; incidents of mass mortality; and the vaccination history of their flocks. The chicken owners were informed of the purpose and context of the study, and their consent was obtained before conducting the interviews.

### Serological analysis

Serum samples were analyzed using the hemagglutination-inhibition (HI) test for the presence of antibodies against Newcastle disease virus (NDV) and low pathogenic avian influenza H9N2 subtype (LPAI H9N2). In addition, we used enzyme-linked immunosorbent assay (ELISA) to screen for the presence of infectious bronchitis virus (IBV) and bursal infectious disease virus (IBDV) antibodies and the rapid serum agglutination (RSA) test to determine the presence of *Mycoplasma gallisepticum* (MG) and *Mycoplasma synoviae* (MS) antibodies.

#### Hemagglutination-inhibition tests

We conducted the HI tests on serum samples using the β procedure (constant antigen and varying serum dilutions) according to the terrestrial manual of the World Organization for Animal Health (WOAH) [10]. We used four hemagglutination units of the lentogenic APMV-1 La Sota strain and A/chicken/Morocco/01/2016 (H9N2) (accession No. KU947112) strains as antigens.

#### Enzyme-linked immunosorbent assay

We used the commercially available ID Screen^®^ IBV Indirect ELISA kit (IDvet, Grabels, France) and PROFLOK^®^ Plus IBD Ab test kit (Zoetis, Florham Park, NJ) according to the manufacturer’s protocols. The results were read using an ELx800 spectrophotometer (BioTek™, Winooski, VT, USA) at 450 nm using the provided software (Gen 5, Biotek, VT, USA).

#### Rapid serum agglutination test

We conducted the RSA test in accordance with the WOAH’s terrestrial manual recommendations [11]. A drop of freshly collected sera was applied with an applicator stick to a white plate, and a drop of commercially available MG- or MS-dyed antigen (Intervet Laboratories, Boxmeer, the Netherlands) was added. The drops were mixed to create a spot with a diameter of 20 mm. The test was read after 2 min of gentle rotation of the plate. Samples that produced visible agglutination were considered positive. To avoid cross-reactions between MG and MS and eliminate false-positive reactions, we diluted the positive sera at (1/5) and decomplemented at 56°C for 30 min, then retested, by mixing 25 μL of the serum with 25 μL of the antigen [11]. During each series of tests, we used the positive and negative control antisera supplied by the manufacturer to check the quality of the antigen.

### Molecular detection of avian pathogens

Cloacal swabs were grouped into 86 pools by grouping three swabs from the same rural market in a common tube containing 5 mL of sterile phosphate-buffered saline. The mixture was then vortexed for a few seconds. We extracted combined RNA/DNA from each pool using the Kylt^®^ RNA/DNA Purification kit (AniCon Labor GmbH, Emstek, Germany) following the manufacturer’s instructions. One-step reverse-transcription–polymerase chain reaction (RT-PCR) was performed to detect viruses using the Brilliant III Ultra-Fast qRT-PCR master mix (Agilent Technologies, Waldbronn, Germany). Table-1 presents the targeted genes, amplicon size, and specific primers and probes of each studied pathogen (IBV, LPAI H9N2, NDV, and IBDV) [12–16]. After adding the master mix to the 96-well plates, 5 mL of sample RNA was added for a total reaction volume of 20 mL. Plates were incubated at 50°C for 30 min, 95°C for 15 min, followed by 40 cycles of 94°C for 15 s, 60°C for 30 s, and 72°C for 30 s. A Ct value of £35 was regarded as positive. We performed polymerase chain reaction (PCR) for MG and MS detection using the Kylt^®^ MGS Triplex real-time (AniCon Labor GmbH, Germany). Any sample with a Ct value of £42 was considered positive.

**Table-1:** Primers and probes used for the detection of viral pathogens.

Pathogens	Target Gene	Amplicon size	Primers and Probe	Reference
NDV	Matrix (M)	121 pb	M+4100 5’AGTGATGTGCTCGGACCTTC-3’ M-4220 5’-CCTGAGGAGAGGCATTTGCTA-3’ M+4169 5’-(FAM) TTCTCTAGCAGTGGGACAGCGC (TAMRA)-3’	[12]
H9N2	Matrix (M)	488 pb	H9f, 5’ ATGGGGTTTGCTGCC 3’ H9r, 5’ TTATATACAAATGTTGC AC (T) CTG3’ Probe, 5’ FAM-TTCTGGGCCATGTCCA ATGG-TAMRA 3’	[13]
IBDV	VP2	474 pb	External primers: Pl primer, 5’- TCACCGTCCTCAGCTTAC-3’ P2 primer, 5’-TCAGGATTTGGGATCAGC-3’ Internal primers: P2.3 (5’-CCCAGAGTC- TACACCATA-3’) P5.3 (5’-TCCTGTTGCCACT-CTTTC-3’)	[14] [15]
IBV	5’ -UTR	143 pb	IBV5 GU391 (5-GCT TTT GAG CCT AGC GTT-3) IBV5 GL533 (5-GCC ATG TTG TCA CTG TCT ATT G-3) G probe (5-FAMCAC CAC CAG AAC CTG TCA CCT C-BHQ1-3	[16]

NDV=Newcastle disease virus, IBDV=Bursal infectious disease virus, IBV=Infectious bronchitis virus

### Statistical analysis

Data from the laboratory analyses were stored in a spreadsheet, and seroprevalence and molecular prevalence were computed. We calculated the prevalence of each disease with 95% confidence intervals (Cls). The prevalence was compared between provinces and rural markets using a chi-square test. A significance level of 5% was used. Analyses were performed using Statistical Package for the Social Sciences 24 (version 24.0, IBM Corp., Chicago, IL, USA). In addition, we summarized responses from the survey regarding the health status and vaccination history of backyard chickens using descriptive statistical analysis.

## Results

According to the interviews with backyard poultry keepers and traders, vaccination against avian diseases was never administered. However, 98.6% of the surveyed persons asserted that they had experienced diseases among their poultry flocks during the previous 6 months, with chickens showing various signs, including depression, inappetence, diarrhea, respiratory distress, and torticollis in some cases.

### Seroprevalence results

Table-2 shows the prevalence of NDV, H9N2, IBD, IBV, MG, and MS antibodies in the surveyed rural markets during the study period. The overall NDV seroprevalence rate was 52.1% (with 95% CI of 46%–71%). Seroprevalence rates of 50% and 62.5% were found in the provinces of Khemisset and Skhirat-Temara, respectively (Table-2), showing a statistically significant difference (p ≤ 0.05) between the two provinces in NDV seroprevalence rates. However, the results obtained indicate that the disease prevailed in 100% of the markets despite fluctuations in seroprevalence (from 27.4% to 100%) and titers (from 4 to 12 Log2) from one sample to another.

**Table-2:** Seroprevalence of NDV, LPAI H9N2, IBDV, IBV, MG, and MS in backyard chickens at rural markets of Khemisset and Temara provinces.

Rural market	No. of tested serum	No. of NDV positive serum (%)	NDV mean titer	No. H9N2 positive serum (%)	H9N2 mean titer	No. of IBDV positive serums (%)	Moyenne des titers ELISA IBD	No. of IBV positive serums (%)	Moyenne des titers ELISA IBV	No. MG positive serums (%)	No. MS positive serums (%)
Brachoua	115	47 (40.9)	5.5	66 (57.4)	5.9	101 (87.8)	4708.7	103 (89.6)	7373.1	69 (60)	93 (81)
Moulay Driss Aghbal	27	10 (37)	4.4	13 (48.1)	6	20 (74.1)	3573	24 (88.9)	2404.5	19 (70.4)	23 (85.2)
Had Lagwalem	25	21 (84)	10.5	14 (56)	6.9	22 (88)	3334.7	20 (80.0)	2501.5	11 (44)	17 (68)
Jemaat Moul Lblad	8	6 (75)	8.6	2 (25)	4	5 (62.5)	6760.8	7 (87.5)	1635.9	5 (62.5)	8 (100)
Ezzhiliga	106	29 (27.4)	4.3	66 (62.3)	6.5	93 (87.7)	3438.3	96 (90.6)	6025.7	70 (66)	90 (84.9)
Rommani	106	49 (46.2)	6.6	75 (70.8)	7.3	101 (95.3)	3577.3	88 (83.0)	5564.1	41 (38.7)	60 (56.6)
Marchouch	156	100 (64.1)	5.8	94 (60.3)	6.1	104 (66.7)	2777.6	110 (70.5)	6770.1	94 (60.3)	102 (65.4)
Ain Sbit	11	11 (100)	6.1	3 (27.3)	7.3	7 (63.6)	2902.1	8 (72.7)	3308	5 (45.5)	8 (72.7)
Sidi Bettach	24	17 (70.8)	7.3	23 (95.8)	9.2	23 (95.8)	4897.8	20 (83.3)	4881.7	14 (58.3)	18 (75)
Houdrane	14	6 (42.9)	3.7	10 (71.4)	6.2	13 (92.9)	7125.6	13 (92.9)	5596.1	9 (64.3)	12 (85.7)
Khemisset province	592	296 (50)^a^	6	366 (61.8)^a^	6.3	489 (82.6)^a^	3797.5	489 (82.6)	4606.1^a^	337 (56.9)^a^	431 (72.8)^a^
Ain Aouda	20	11 (55)	7.2	14 (70)	6.4	16 (80)	5278.7	14 (70.0)	4018.1	11 (55)	16 (80)
Sidi Yahia Zaer	21	7 (33.3)	7.6	16 (76.2)	8.4	21 (100)	5167.8	17 (81.0)	5459.1	13 (61.9)	19 (90.5)
Mers El Khier	28	20 (71.4)	7.2	19 (67.9)	6.9	28 (100)	3950	24 (85.7)	5260	23 (82.1)	26 (92.8)
Hadd Sebbahe	20	8 (40)	6.9	14 (70)	6.9	19 (95)	3496.2	13 (65.0)	4179.7	8 (40)	17 (85)
Larbaa Ain Atiq	31	29 (93.5)	10.8	23 (74.2)	7.4	30 (96.8)	3097.9	28 (90.3)	4785.2	21 (67.7)	24 (77.4)
Temara province	120	75 (62.5)^b^	8.5	86 (71.7)^b^	7.2	114 (95)^b^	4063.7	96 (80.0)	4740.42^a^	76 (63.3)^a^	102 (85)^b^
Total	712	371 (52.1)	6.5	452 (63.5)	6.5	603 (84.7)	3860.6	585 (82.2)	4650.8	413 (58)	533 (74.8)

Different superscript letters in the same row indicate a significant difference (p<0.05). NDV=Newcastle disease virus, IBDV=Bursal infectious disease virus, IBV=Infectious bronchitis virus, MG=*Mycoplasma gallisepticum*, MS=*Mycoplasma synoviae,* ELISA=Enzyme-linked immunosorbent assay, LPAI H9N2=Low pathogenic avian influenza H9N2 subtype

The seroprevalence of LPAI H9N2 and the mean IHA titer of anti-H9N2 antibodies recorded in the present study were 63.5% (95% CI: 53%–72%) and 7.4 Log^2^, respectively. The province of Skhirat-Temara showed the highest rate of H9N2 seroprevalence of 71.7% against 61.8% noted in the province of Khemisset. Chi-square test showed a statistically significant difference between the two areas (p ≤ 0.05). The lower prevalence value observed in the rural markets was 25%, whereas the upper prevalence was almost 96%.

Of the 712 serum samples collected, 603 tested positive for IBDV, resulting in an overall prevalence of 84.7% (95% CI: 79%–92%) in the study area. In the Khemisset and Skhirat-Temara provinces, the prevalences were 82.6% and 95%, respectively, with statistically significant differences (p ≤ 0.05). The seroprevalence of Gumboro disease in the Skhirat-Temara region varied from 80% to 100%, whereas slightly lower seroprevalences were observed in the Khemisset province. Values ranged from 62.5% to 100%. In addition, most titer values (69%) were between 3000 and 18,500.

Of the 712 chickens sampled, 82.2% (95% CI: 77.4%–86.4%) were positive for IBV antibodies, 82.6% (489/592) and 80% (96/120) in Khemisset and Skhirat-Temara, respectively (Table-2). For *Mycoplasmas*, Of the 712 serum samples tested by RSA test, 413 and 533 were found to be positive for MG and MS, respectively, showing an overall prevalence of 58% (95% CI: 52%–65%) and 74.8% (95% CI: 74%–86%).

### Molecular detection of avian pathogens

Of the 86 pooled swab samples tested by real-time RT-PCR and PCR, we identified 2.3% (2/86) of the samples as positive for NDV in the study area. H9N2 was detected in 62.8% (54/86) of the pooled swabs sampled from backyard chickens in rural markets. Regional variations were observed in H9N2 detection. Khemisset province had significantly higher H9N2 detection (p < 0.05) than Skhirat-Temara province (Table-3). Overall, IBD and IBV were detected in 2.3% (2/86) and 63.9% (55/86) of the tested pools, respectively. Similarly, there were significant (p < 0.05) differences in IBV detection between the two provinces, with a lower value found in Skhirat-Temara province and a higher value in Khemisset (Table-3). Our analysis also found that 40.7% and 29.1% of the tested pools were positive for MG and MS, respectively.

**Table-3:** Pathogens detection in pooled swabs from backyard chickens in rural market of Khemisset and Temara provinces.

Rural market	No. analyzed swabs	No. of pools	NDV	H9N2	IBDV	IBV	MG	MS
Brachoua	27	9	0/9	7/9	1/9	5/9	4/9	1/9
Moulay Driss Aghbal	15	5	0/5	4/5	3/5	3/5	3/5	5/5
Had Lagwalem	9	3	0/3	2/3	0/3	3/3	2/3	2/3
Jemaat Moul Lblad	12	4	1/4	2/4	0/4	4/4	3/4	2/4
Ezzhiliga	15	5	0/5	5/5	0/5	5/5	3/5	1/5
Rommani	36	12	0/12	11/12	0/12	6/12	7/12	1/12
Marchouch	33	11	0/11	5/11	0/11	7/11	4/11	2/11
Ain Sbit	30	10	0/10	2/10	0/10	2/10	2/10	1/10
Houdderane	9	3	0/3	3/3	1/3	1/3	1/3	0/3
Sub-total	186	62	1/62	41/62	2/62	36/62	29/62	15/62
Percentage (%)			1.6	66.1	3.2	58.1	46.8	24.2
Ain Aouda	9	3	0/3	1/3	0/3	3/3	0/3	2/3
Sidi Bettach	9	3	0/3	1/3	0/3	2/3	2/3	1/3
Sidi Yahya Zaeir	6	2	0/2	2/2	0/2	2/2	1/2	1/2
Mers El Khier	12	4	0/4	2/4	0/4	4/4	1/4	0/4
Sebbahe	9	3	0/3	2/3	0/3	1/3	1/3	0/3
Ain Atiq	27	9	1/9	5/9	0/9	7/9	1/9	6/9
Sub-total	72	24	1/24	13/24	0/24	19/24	6/24	10/24
Percentage (%)			4.2	54.2	0	79.2	25	41. 7
Total			2/86	54/86	2/86	55/86	35/86	25/86
Percentage (%)			2.3	62.8	2.3	63.9	40.7	29.1

NDV=Newcastle disease virus, IBDV=Bursal infectious disease virus, IBV=Infectious bronchitis virus, MG=*Mycoplasma gallisepticum*, MS=*Mycoplasma synoviae*

## Discussion

Investigations of the health status of backyard chickens during periods without epidemics provide important information on the circulation of low-noise diseases for backyard flock owners, industry poultry farms, and state animal health decision-makers. The positive serological results prove that the unvaccinated birds had been exposed to the infectious agents under inquiry. In this study, the chicken owners confirmed that they had never vaccinated their birds against any poultry disease. Therefore, antibodies against ND, H9N2, IBD, IBV, MG, and MS were viewed as proof of natural exposure to infection.

Our study revealed that the prevalence of NDV in backyard chickens was slightly high by HI test at 52.1% and low by RT-PCR at approximately 4%. A study published in 1988 reported the exposure of backyard chickens to NDV in Morocco, with an estimated overall ND seroprevalence of 35.2% in six different regions [17]. In addition, two virus isolates from each region were isolated, characterized, and found to be velogenic. Thus, the authors concluded that backyard chicken flocks represent a reservoir of virulent NDV throughout Morocco. Similar seroprevalence results were reported in neighboring countries such as Libya, where the seroprevalence was approximately 53% [18], and in Senegal, with a seroprevalence of 54.4% was reported in rural chickens [19]. Many researchers worldwide have highlighted the prevalence of NDV in backyard chickens and its implication as a serious threat to the industrial sector and other avian species [20, 21]. In addition, the fact that >60% of the sera tested have antibody titers between 4 and 7 Log 2, whereas 34% of the sera showed higher titers (>7 Log2), suggests that the circulation of lentogenic or mesogenic NDV strains continuously maintains a medium level of antibodies [17, 22]. The high NDV HI antibody titers point to velogenic NDV outbreaks that might have killed most chickens in some villages and left only a small number of survivors with high antibody titers [23, 24].

Furthermore, the very low prevalence of NDV detected by RT-PCR in the tested samples demonstrates the absence of recent or current virus infection in most of the sampled chickens. Various studies have reported positive serology and negative RT-PCR results for NDV. In Oman, 2,350 domestic birds were tested, and RT-PCR detected no cases of NDV [25]. In Brazil, among 292 cloacal and orotracheal samples tested by RT-PCR, 87.5% seroprevalence was found, but no positive results were obtained [26]. In New Zealand, the virus was not detected in any of the 162 cloacal and orotracheal pools collected from seropositive hens [27]. These negative results confirmed the absence of recent or current NDV infections in the sampled backyard chickens. All chickens were healthy, with no apparent clinical signs at time of sampling.

The serological and molecular results indicated that the LPAI H9N2 virus was present in backyard chickens with high prevalence of 63.5% and 62.8% using the HI test and RT-PCR, respectively. These high prevalence levels confirm the endemic nature of this disease in backyard poultry flocks in Morocco. Since its first detection in broiler poultry farms in 2016 [28], LPAI H9N2 virus has circulated among wild birds and commercial poultry flocks, resulting in substantial economic losses. Despite control efforts, the H9N2 virus continues to spread among commercial poultry in Morocco [29]. Moreover, the presence of LPAI H9N2 infection among the backyard chicken population reported in our study makes it an additional risk factor for the poultry industry. It indicates that this population might represent the transmission of the virus between wild birds and domesticated birds in both directions [30]. Furthermore, the ability of LPAI H9N2 to infect and be transmitted to both chickens and humans highlights the importance of characterizing the circulating strains in poultry. Also, it requires continuous surveillance of influenza virus infections [29, 31].

The IBV prevalence observed in this study averaged 82.2% and 63.9% by ELISA and RT-PCR, respectively. Little information is available on the IBV prevalence in backyard poultry flocks in Morocco. Investigations on IBV are predominantly focused on the commercial poultry sector. Since the first isolation and characterization of the virus in Morocco in 1985 [32], several outbreaks of IBV have been reported in layers, breeders, and broilers. In a study conducted in 2015, the authors reported a prevalence of IBV detection by RT-PCR of up to 51.7% in 360 chickens with clinical signs of respiratory distress, renal lesions, decline in egg production, and damage to egg shape [33]. Since then, no studies have estimated the prevalence of IBV in the population of backyard chickens. Our results demonstrate for the first time the high levels of IBV occurrence in backyard chickens, as shown also for commercial flocks in other studies. The IBV seroprevalence detected in our study was 82.2%, which is much higher than the seroprevalence of NDV and H9N2, whereas the prevalence of the viral genomes was 63.9%, similar to the H9N2 molecular prevalence of 62.8% and higher than the NDV prevalence. Our results are in agreement with the previous studies conducted by Kouakou *et al*. [34] and Tesfaye *et al*. [35] in Ivory-Coast and Ethiopia, which also found high levels of IBV circulation in live backyard chickens at the market level, with a seroprevalence >70% [34, 35].

The results of the present study revealed a very high seroprevalence (84.7%) of IBD in unvaccinated backyard chickens, indicating evidence of virus circulation and subsequent field exposure of chickens. Indeed, Gumboro disease has been endemic in Morocco since it was first detected in commercial poultry flocks in 1978. Recently, the highly virulent strain very virulent IBDV has been isolated and characterized in many regions of the country, demonstrating that this strain, which is distinct from classic and vaccine strains, is widespread [36]. Our observation of a high seroprevalence of IBDV in this backyard production system is in agreement with previous serological studies conducted in different countries. A study conducted in Santa Maria, Ecuador, reported a high seroprevalence of IBD of up to 100% in backyard chickens [37]. Similarly, an overall prevalence of 66.9% was reported in Western Oromia, Ethiopia [38], whereas a high seroprevalence of 76% was reported in backyard chickens in the Amman area [39]. In contrast, other studies have suggested a relatively lower prevalence, such as 33.4% reported in Maiduguri, Nigeria [40]; 30.6% in Sudan [41]; and 34% in Pakistan using the agar gel immunodiffusion assay (AGID) test [42]. The disparity in our findings might be due to the differences in the testing methods used. Serology results can vary depending on the sensitivity and specificity of the implemented diagnostic tool [43], and ELISA tests are known to be more sensitive than AGID [44]. Although in our study, there was a high seroprevalence of Gumboro disease detected by ELISA (84.7%), only 2% of the tested pools were positive by quantitative RT-PCR. The absence of the IBD virus in the swab samples does not exclude its circulation among the backyard chicken population. However, this might be due, first, to the nature of the samples originally tested, which were cloacal swabs. However, the virus was diagnosed mainly in the bursa of Fabricius, which is the main target organ for the virus [45]. Second, it might also be due to the nature of the virus, which is difficult to isolate and identify, making the most practical diagnosis the detection of specific antibodies to the virus [44].

Several pathogenic mycoplasmas cause avian mycoplasmosis, the most important of which are MG and MS. Both MG and MS can cause respiratory diseases and synovitis in poultry, resulting in severe economic losses. Antibodies against MG and MS were found in 58% and 74.8% of the chickens by RSA and in 40.7% and 29.1% of the pools tested by RT-PCR, respectively (Tables-2 and 3). Several pathogenic mycoplasmas cause avian mycoplasmosis. These findings indicate that backyard poultry farming systems could play a significant role in spreading such pathogens to the commercial poultry sector, particularly in regions where poultry density is high. The present finding regarding MG seroprevalence is in agreement with the results of several previous reports from Ethiopia (57.7%) [22], Bangladesh (54.9%) [46], and Mongolia 53.0% [47]. A higher prevalence of MG was reported in backyard chickens in Ecuador (73%) [37] and South Africa [48]. A lower MG seroprevalence result of 35% was obtained from backyard chickens in Pakistan [49]. In Morocco, researchers investigated the prevalence of MG and MS in broiler chickens from 1983 to 2005 [50]. The results of serological analysis by RSA showed an increase in rates of infection from 2.10% for MG and 59.5% for MS to 26.67% and 66.67%, respectively. Other studies based on the molecular detection of MG and MS have demonstrated their circulation in village chickens in several countries. In Italy, for example, 45.4% of the sampled backyard chicken flocks were found to be positive for MG and MS [51], whereas higher prevalences were recorded in Costa Rica (29% for MG and 67% for MS) [52]. The present results confirm the circulation of MG and MS among the backyard chicken population in Morocco and its potential role in spreading pathogens to commercial poultry settings. In terms of coinfection, 3.5% of the pools (3/86) tested positive for four pathogens, 34.9% (30/86) for three pathogens, and 31.4% (27/86) for two pathogens. On the other hand, 26.7% of the pools (23/86) did not present any coinfection, and 3.5% (3/86) were negative for all infectious agents detected by PCR. Because each pool contained material from three birds, this result could mean that the multiple pathogens either came from distinct birds or that a single bird had multiple infections. Among the avian pathogens, coinfection with H9N2-IBV-MG is the most encountered combination (15.1%), followed by coinfections with H9N2-IBV-MS or IBV-MG-MS, at 8.5% each.

Despite the great importance of backyard poultry farming to a large proportion of the rural population in Morocco, as in many other developing countries, and its potential implication as a serious threat to the industrial sector, to the best of our knowledge, very few studies have been performed on the presence of poultry diseases among the backyard chicken population in Morocco. Within this context of a lack of information, our finding constitutes the first investigation on the disease prevalence within the backyard chicken population in this country. The results of our study provide evidence of a high prevalence of avian diseases in backyard chickens, which has the potential to serve as a reservoir or amplifier of avian diseases that might affect commercial poultry [53, 54]. The high prevalence detected in our study is chiefly the result of the extensive farming system characterized by poor sanitary conditions, continuous exposure of chickens to free-range environments and wild birds, and bringing together different species and ages in the same place, all of which, due to the lack of any vaccination and biosecurity measures, increase the transmission of disease between birds. Furthermore, this finding indicates the significant role of the rural market in avian disease epidemiology. Contact between birds with unknown health status, transported from various areas to rural markets, can undoubtedly facilitate the rapid spread and persistence of an endemic pattern of avian diseases. Transporting birds across long distances, from farms to markets, in stressful conditions can lead to immunodepression, which obviously increases the susceptibility to pathogens [55]. Therefore, due to the existence of infection in backyard poultry and the potential transmission of infection to commercial poultry farms, it is imperative to include backyard poultry in the disease surveillance system and control strategy. Furthermore, outreach education programs addressed to backyard poultry keepers, notably regarding the implementation of biosecurity measures at the farm level, along with an effective and adapted vaccination program, can play an important role in reducing the occurrence and effects of major poultry diseases in backyard poultry [56, 57].

## Conclusion

This study provided evidence that the most feared pathogens in commercial poultry farms are circulating among backyard chicken populations. The epidemiological figures in this study highlight the need for an effective and adapted avian disease prevention and control strategy for backyard chickens in Morocco. In addition, great efforts should be made in terms of awareness, extension, and education of flock owners about avian diseases. Implementation of biosecurity measures in farms should improve the epidemiological situation, productivity, and income of small farmers. On the other hand, backyard poultry flocks have proven to be good sentinels of avian pathogens in a given environment. Therefore, it is imperative to consider their relevance for any avian disease surveillance system. Most importantly, exhaustive studies should be conducted to isolate and identify pathogen strains and investigate their molecular profiles to better understand the potential epidemiological role of strains circulating among backyard chicken populations.

## Authors’ Contributions

AF, MB, and SF: Designed the study. AF, OA, and OK: Collected the samples. AF, OA, OK, and FZE: Conducted the analysis. AF, MB, SF: Interpreted results. AF: Wrote the manuscript. MB, FK, and SF: Reviewed the manuscript. All authors have read and agreed to the published version of the manuscript.
